# Association between dietary inflammatory index score and muscle mass and strength in older adults: a study from National Health and Nutrition Examination Survey (NHANES) 1999–2002

**DOI:** 10.1007/s00394-022-02941-9

**Published:** 2022-07-09

**Authors:** Lingzhi Chen, Jingjing Ming, Tianyi Chen, James R. Hébert, Peng Sun, Li Zhang, Hongya Wang, Qingkuo Wu, Cancan Zhang, Nitin Shivappa, Bo Ban

**Affiliations:** 1 Department of Clinical Nutrition, Affiliated Hospital of Jining Medical University, Jining, 272029 Shandong China; 2 Department of Emergency Trauma Surgery, Jining No. 1 People’s Hospital, Jining, 272029 Shandong China; 3 School of Nursing, Shandong First Medical University, Shandong Academy of Medical Sciences, Tai’an, 271000 Shandong China; 4 Department of Biochemistry and Molecular Biology, Harbin Medical University, Harbin, 150081 Heilongjiang China; 5 Cancer Prevention and Control Program, University of South Carolina, 915 Greene Street, Suite 200, Columbia, SC 29208 USA; 6 Department of Epidemiology and Biostatistics, University of South Carolina, 915 Greene Street, Suite 400, Columbia, SC 29208 USA; 7 Department of Nutrition, Connecting Health Innovations, LLC, 1417 Gregg Street, Columbia, SC 29201 USA; 8 Department of Clinical Nutrition, Yan Tai Yu Huang Ding Hospital, Yantai, 264000 Shandong China; 9 Department of Obstetrics, Affiliated Hospital of Jining Medical University, Jining, 272029 Shandong China; 10 Department of Clinical Nutrition, Tumor Hospital of Jining, Jining, 272029 Shandong China; 11 Department of Endocrinology, Affiliated Hospital of Jining Medical University, Jining, 272029 Shandong China; 12 Chinese Research Center for Behavior Medicine in Growth and Development, Jining, 272029 Shandong China

**Keywords:** Dietary inflammatory index, Appendicular skeletal muscle index, NHANES, Low muscle mass, Low muscle strength

## Abstract

**Purpose:**

Chronic low-grade systemic inflammation affects muscle protein metabolism. The dietary inflammatory index (DII®) is a tool designed to assess the inflammatory potential of the diet. The available data on the association between DII and sarcopenia are limited. We aimed to investigate the association of the DII with components of sarcopenia in individuals over 50 years of age.

**Methods:**

This cross-sectional study used the National Health and Nutrition Examination Survey (NHANES) 1999–2002 dataset. Body composition was measured, and isokinetic strength of the knee extensors (peak force) was evaluated. Low muscle mass and strength were defined using sex-specific thresholds. Energy-adjusted DII (E-DII™) scores were calculated using 24-h dietary recall data. Regression models were fit to evaluate the association between E-DII scores and low muscle mass and low muscle strength, alone and combined.

**Results:**

Mean age of study participants was 62.1 ± 9.5 years, and 138 participants (7.4%) belonged to the combination group of low muscle mass and low muscle strength. In multivariable-adjusted regression models, higher E-DII score was associated with lower appendicular skeletal muscle index (ASMI) (*β* =  − 0.03, *P*  < 0.001, *P* trend <0.001), and lower peak force (β =  −2.15, *P * = 0.04, *P* trend = 0.01) and higher likelihood for these components combined (OR = 1.12, 95% CI 1.01–1.25, *P* = 0.03).

**Conclusion:**

Higher E-DII score is associated with lower muscle mass and muscle strength, and increased likelihood for the combination of low muscle mass and low muscle strength in older adults. This has important implications for healthy aging.

**Supplementary Information:**

The online version contains supplementary material available at 10.1007/s00394-022-02941-9.

## Introduction

Muscle plays an important role in protein metabolism [1]. Skeletal muscle, the largest organ in the body by mass, is responsible for 80% of postprandial glucose uptake from the circulation [2]. Considering its role in glucose uptake and its importance in exercise and metabolic disease, skeletal muscle is critical for metabolism [3]. Skeletal muscle also plays an important role in peripheral insulin resistance [3]. Muscle-secreted cytokines (myokines) exert endocrine effects on metabolic regulation [4]. Skeletal muscle also is a primary source of ROS production during exercise [5]. There is a balance between the processes of synthesis and degradation, which is dependent on the continuous renewal of muscle proteins [6]. Regulation of protein content is achieved through alterations of the synthesis rates as the primary response; increases in the breakdown rate during net catabolism appear to occur only in response to exceptional situations (such as starvation) [7]. The mammalian target of the rapamycin (mTOR) signaling pathway plays a critical role in regulating protein synthesis to maintain muscle protein turnover and trophism [8]. Altered muscle metabolism is a significant determinant of acute illness (such as sepsis and traumatic injury) and chronic diseases (such as obesity and diabetes) [1].

In younger populations, muscle mass is known to be inversely related to cardiometabolic risk and metabolic syndrome [9, 10]. Muscular strength has been shown to have an independent protective effect on all-cause and cancer mortality in healthy middle-aged men, as well as in men with cardiovascular disease (CVD) [11]. A strong association between muscle mass and muscle strength exists. Several molecular mechanisms have been described as causes for degenerative loss of skeletal muscle mass, quality, and strength [12]. These mechanisms include, for example, the function of hormones (e.g., IGF-1 and Insulin), muscle fiber composition and neuromuscular drive, myo-satellite cell potential to differentiate and proliferate, inflammatory pathways as well as intracellular mechanisms in the processes of proteostasis and mitochondrial function [13]. Optimizing diet and nutrition status during the life cycle may be an important strategy to prevent sarcopenia [14]. A systematic review provides evidence of the beneficial effect of dairy protein as a potential nutrition strategy to improve appendicular muscle mass in middle-aged and older adults [15].

Loss of muscle mass and related function is a feature typically exhibited by people with chronic diseases as well as during aging [16]. Age-related musculoskeletal conditions, which includes fragility fractures, low muscular mass and strength, and frailty, are associated with chronic inflammation [17, 18]. Chronic inflammation, characterized by elevated concentrations of mediators such as IL-6, TNFα and C-reactive protein (CRP), is associated with lower muscle mass and strength in the elderly population [19].Chronic low-grade systemic inflammation affects muscle protein metabolism through multiple signaling pathways, leading to loss of muscle mass, strength, and function [20]. Although the origin of sarcopenia is multifactorial, ranging from aging and cancer-related mechanisms, the role of chronic systemic inflammation is associated with the onset of skeletal muscle alterations. Its role in the context of metabolic syndrome requires additional considerations as impaired insulin activity in skeletal muscle is responsible for the altered molecular pathways and clinical manifestations of sarcopenia [21].Weight loss is a critical factor to reduce inflammation, and the effect can be related to body fat loss [22].

Diet may play an important role in the regulation of chronic systemic inflammation [23, 24] and possibly in muscle fitness. The dietary inflammation index (DII®) estimates the overall inflammatory potential of a diet based on the pro-inflammatory and anti-inflammatory properties of various dietary components (food parameters) [25]. Higher DII scores indicate a greater dietary inflammatory potential, which is reflected in higher plasma levels of inflammatory biomarkers [26, 27]. Research results in Australian community-dwelling older men showed that a higher energy-adjusted DII (E-DII™) score was associated with lower appendicular lean mass after adjusting for BMI (body mass index); while E-DII was not associated with changes in handgrip strength over 3 years [18]. The cross-sectional study in older Australians from the Geelong Osteoporosis Study showed that a pro-inflammatory diet was associated with poorer muscle function, which was defined as Timed-Up-and-Go >10 s over 3 m [28]. In Australian community-dwelling older adults, it was shown that pro-inflammatory diets may be more harmful to musculoskeletal health in men than in women [29]. A population-based cross-sectional study including 300 elderly Iranians revealed that a diet with more pro-inflammatory potential was associated with a greater odds of sarcopenia. However, no significant association was seen between DII and components of sarcopenia including low muscle mass and abnormal handgrip strength [30]. In early 2018, the European Working Group on Sarcopenia in Older People (EWGSOP) produced an updated consensus paper on sarcopenia, and used detection of low muscle quantity and quality to confirm the sarcopenia diagnosis [31]. In our study, a combination of low muscle mass and low muscle strength was used as a representation of sarcopenia. Low muscle mass was defined using the Foundation for the National Institute of Health definitions: ASM divided by BMI (men <0.789; women <0.512) [32, 33]. Low muscle strength was defined as the peak torque value that fell more than 2 SDs below the sex-specific mean peak torque of this healthy 50–59-year-old reference group (men <104.4 Nm; women <62.6 Nm) [34].

Evidence on the association between the DII and muscle fitness is very limited among older Americans. Hence, the aim of this study was to investigate the association between the inflammatory potential of diet and skeletal muscle mass and strength and a combination of these two as a representation of sarcopenia in older US adults.

## Methods

### Research design and population

This research is based on the 1999–2002 NHANES, a cross-sectional survey conducted by the United States Centers for Disease Control and Prevention, which initiated this survey in 1971. Survey contents and procedure manuals can be found at https://www.cdc.gov/nchs/nhanes/index.htm (accessed March 2021). This survey represents non-institutionalized community-dwelling residents in the United States and, as such, uses multistage, stratified, complex probability sampling designs and oversamples specific groups such as race/minority groups and seniors. Due to the de-identified nature of the data analyzed, this study was exempt from review by our local institutional review board.

NHANES 1999–2002 screened a total of 25,316 participants, 21,004 of whom were interviewed. Respondents with body composition measures (*n* = 11,096) were included. Participants who had BMI <18.5 kg/m^2^ were excluded (*n* = 1257). Participants with missing E-DII score were excluded (*n* = 265). Furthermore, participants who reported values for total energy intake outside of the predefined limits (<3347 kJ (<800 kcal)/d or >17,573 kJ (>4200 kcal)/d for men; < 2510 kJ (<600 kcal)/d or >14,644 kJ (>3500 kcal)/d for women) were excluded (*n* = 1126). These limits were set in accordance with those recommended by Willett in Nutritional Epidemiology [35]. The muscle strength exam selected subjects ≥50 years of age for the test. Participants aged <50 years were excluded (*n* = 5690). Subjects with extreme values of peak force velocity (>65 degrees/second or <55 degrees/second) were excluded (*n* = 882) [36, 37]. Participants who did not perform at least four trials in the strength test were excluded (*n* = 13). Ultimately, the overall study population included 1863 participants.

## Dietary inflammatory index and muscle measures

The 24-h dietary recall interview (24HR) was conducted by trained staff at the mobile examination center (MEC). This format asked participants to recall all foods and beverages consumed in a 24-h period the day before the interview. The development and validation of the DII have been shown in detail elsewhere [25, 26, 38]. The DII score was calculated using the information derived from the 24HR. The DII food parameters available in the NHANES database included carbohydrates; protein; fat; alcohol (grams); fiber; cholesterol; saturated, monounsaturated, and polyunsaturated fatty acids; n-3 and n-6 polyunsaturated fatty acids; vitamins A, B1, B2, B3, B6, B12, C, D, and E; iron; magnesium; zinc; selenium; folic acid; *β*-carotene; and caffeine. Higher (i.e., more positive) scores indicate more pro-inflammatory diets and more negative values are more anti-inflammatory [25]. The energy-adjusted DII (E-DIITM) was expressed per 1000 cal consumed and was computed in a manner analogous to the DII, but utilized an energy-adjusted global comparative database.

All body composition measures were performed using whole-body DEXA (dual-energy X-ray absorptiometry) scans (Hologic Scanner, QDR-4500, Bedford, MA). This assessment excluded individuals with a height of >192.5 cm or a weight of >136.4 kg. All metal, excluding false teeth and hearing aids, was removed prior to assessment. All non-fat and non-bone mass were considered skeletal muscles, and appendicular skeletal muscle mass (ASM) was defined as the sum of the lean soft tissue from the limbs. We quantified muscle mass using the appendicular skeletal muscle index (ASMI), calculated as the ASM divided by the square of the height [39]. Voluntary peak isokinetic knee extensor strength was evaluated using a Kinetic Communicator isokinetic dynamometer (Kin Com MP, Chattecx Corporation, Chattanooga, TN). Participants performed 6 measurements of muscle strength of the right quadriceps at an angular velocity of 60 degrees/sec. Subjects with extreme values of peak force velocity (>65 degrees/sec or <55 degrees/sec) were excluded [36, 37]. Each subject would have a total of six trials during the strength test, and the first three trials were practice warm-ups. In the last three trials, they were strongly encouraged to perform at maximal effort. The individuals with fewer than four trials were excluded. Highest peak force (PF) in Newtons was obtained from the maximum effort trials for those who completed at least four trials. Peak torque (PT) was calculated as: ${{\left( {{\text{PF}} \times {\text{mechanical arm length in centimeters}}} \right)} / {100}}$ [34, 36]. Individuals who presented with a history of myocardial infarction within the past 6 weeks, or had knee surgery or knee replacement surgery, chest or abdominal surgery within the past 3 weeks, a history of brain aneurysm or stroke, or severe back pain did not perform the muscle strength evaluation.

## Covariates

Respondents were surveyed on demographic variables, including age, sex, race/ethnicity, marital status, place of birth, and education. Marital status was categorized as married and unmarried, and unmarried includes never married, separated, divorced, or widowed. Self-reported educational attainment was categorized as less than high school, high school, or more than high school. Health status (complications, yes/no) and health behaviors of the respondents were evaluated via questionnaires. When the respondent was “informed by a doctor or other health professional” of comorbidities, the study defined the respondent as having comorbidities. The chronic disease complications in this study included diabetes, hypertension, coronary artery disease (CAD), congestive heart failure (CHF), angina, heart attack, stroke, cancer, and renal failure. Chronic disease (yes/no) was defined as follows: participants who do not have any of the 9 diseases named above were defined as having "no chronic diseases"; participants with one or more of the above 9 diseases were defined as having “chronic disease(s)”. Physical activity was categorized in four levels (sits, walks, light loads, and heavy work) using a self-reported questionnaire that asked participants “Please tell me which of these four sentences best describes your usual daily activities (sits: sits during the day and does not walk about very much; walks: stand or walk about a lot during the day but does not have to carry or lift things very often; light loads: lifts a light load or has to climb stairs or hills often; heavy work: does heavy work or carries heavy loads)”. Participants were classified as never smokers, former smokers or current smokers. BMI was calculated as the weight (kg) divided by the square of the height (m)^2^. Height and weight were measured and recorded according to NHANES anthropometric standards.

## Statistical analysis

All the data were combined into one dataset according to the NHANES protocol, and data analyses accounted for the masked variance and used the recommended weighting methodology. First, we grouped the E-DII into tertiles and assessed the baseline characteristics of participants. The continuous variables are expressed as the means with their standard errors (with groups compared via ANOVA). The categorical variables are expressed as percentages (with groups compared using the *χ*^2^ test). We calculated the Pearson correlation coefficients between E-DII and nutrient intake (total energy, macronutrients, *ω*-3 fatty acids). We used linear regression models to examine associations between muscle fitness and demographic characteristics and lifestyle and nutrient variables. The primary goal was to determine the association between E-DII (predictor) and muscle mass and muscle strength and these components combined (outcome); therefore, the results of the models that were not adjusted and those adjusted for potential confounders are presented based on the recommendations of the STROBE statement. When the covariate was added to the model and the odds ratio changed by at least 10%, the covariates were deemed needed to be adjusted [40].

In the association analyses, the E-DII was first analyzed as a continuous variable and then classified into three groups (tertiles) to facilitate interpretation. To further explain the association between E-DII score (predictor) and muscle mass and muscle strength (outcome), ASMI was first selected for linear regression, and then ASM/BMI (continuous variable) was classified into low versus preserved muscle mass according to clinical significance. In terms of muscle strength, peak force (PF) was first selected for linear regression, while low muscle strength was defined by peak torque sex-specific value. A logistic regression model was used to examine the association between DII score and low muscle mass and low muscle strength and these components combined (sarcopenia). ASMI and PF regression coefficients and 95% confidence intervals (CIs) were evaluated by constructing a series of hierarchical models that adjusted for potential confounders: Model 1, adjusted for age, sex, race, education, marital status, nativity, smoking, physical activity level and BMI; Model 2, additionally adjusted for chronic disease, energy and protein. Logistic regression models, using a similar adjustment strategy, were then used to derive the OR and 95% CI for the association of E-DII (by tertile) with low muscle mass and low muscle strength and sarcopenia, using the lowest tertile as a reference. Serum vitamin D data were available in the NHANES 2001–2002 cycle, and it is necessary to consider vitamin D as an important potentially confounding factor. The multiple linear regression model further adjusted the content of vitamin D in the concentrated sample, and whether the association exists stably. Malnutrition and physical inactivity are the main risk factors of skeletal muscle loss contributing to the onset of sarcopenia [41]. Subgroup analyses were performed based on nutritional status and exercise status.

All analyses were performed using the statistical software package R (http://www.R-project.org, The R Foundation) and EmpowerStats (http://www.empowerstats.com, X&Y Solutions, Inc., Boston, MA). *P* values less than 0.05 (two sided) were considered statistically significant.

## Results

### Baseline characteristics of participants

Characteristics of the 1863 participants included in the analyses are shown in Table 1. The average age of the participants was 62.1 ± 9.5 years, and slightly over half (51.0%) were female. The E-DII score ranged from −5.79 to +4.39, with a mean of −0.16 (SE: 1.98). The average ASMI of the participants was 7.26 ± 1.31 kg/m^2^, and the ASMI (male vs. female) was 8.14 vs. 6.42 kg/m^2^. The average peak force of the participants was 377.0 ± 121.9 Newtons, and the peak force (male vs. female) was 450.9 vs. 305.8 Newtons. A total of 138 participants (7.4%) of the entire population belonged to the combination group of low muscle mass and low muscle strength; 1290 participants (69.2%) belonged to the normal muscle mass and normal muscle strength status group. A total of 223 participants belonged to the low muscle mass group, accounting for 12.0% (223/1863); and the remaining 212 participants belonged to the low muscle strength group, accounting for 11.4% (212/1863).

**Table 1:** Weighted demographic characteristics, anthropometric and body composition, and strength data of the participants by tertile of energy-adjusted dietary inflammatory index (E-DII)

	E-DII score category				
	Total	T1 ^a^	T2	T3 ^b^	*P*-value
Min and max of DII	−5.79 to 4.39	−5.79 to −1.10	−1.09 to 0.77	0.77 to 4.39	
Participants, *n*	1863	621	621	621	
DII score	−0.16 ± 1.98	−2.47 ± 1.05	−0.17 ± 0.55	1.93 ± 0.76	<0.001
Demographic data					
Age (years)	62.1 ± 9.5	63.8 ± 9.9	62.4 ± 9.5	60.2 ± 8.6	<0.001
Sex (%)					<0.001
Male	49.0	41.8	47.5	57.1	
Female	51.0	58.2	52.5	42.9	
Race (%)					0.03
Non-Hispanic White	81.3	83.7	79.1	81.3	
Non-Hispanic Black	6.8	4.8	6.5	8.8	
Mexican American	3.5	3.4	3.7	3.4	
Other Hispanic	5.2	4.3	7.2	4.0	
Other Race	3.2	3.7	3.6	2.5	
Education (%)					<0.001
Less than high school	21.8	15.8	23.5	25.7	
High school diploma	25.6	23.1	26.7	26.8	
More than high school	52.6	61.1	49.9	47.5	
Marital status (%)					0.69
Unmarried	25.1	25.9	25.6	23.9	
Married	74.9	74.1	74.4	76.1	
Nativity (%)					<0.001
Foreign-born	11.9	15.2	12.7	8.0	
US-born	88.1	84.8	87.3	92.0	
Lifestyle variables					
Smoking (%)					< 0.001
No	45.0	53.5	43.0	39.2	
Former smoker	39.2	39.9	42.4	35.7	
Current smoker	15.8	6.6	14.6	25.0	
Physical activity level (%)					0.50
Sits	23.7	25.0	22.4	23.7	
Walks	55.3	54.5	56.8	54.5	
Light loads	16.6	17.0	16.8	16.1	
Heavy work	4.4	3.5	3.9	5.7	
Comorbidities					
Diabetes (%)	10.7	11.3	10.2	10.5	0.80
Hypertension (%)	39.5	40.8	42.2	35.7	0.04
Coronary artery disease (%)	6.3	5.6	8.0	5.3	0.10
Congestive heart failure (%)	2.2	2.0	2.8	1.9	0.52
Heart attack (%)	5.2	5.7	6.3	3.8	0.10
Angina (%)	5.3	5.7	5.7	4.4	0.50
Stroke (%)	0.7	0.7	1.3	0.1	0.03
Cancer (%)	14.7	15.7	17.5	11.1	0.004
Renal failure (%)	1.8	1.4	2.0	2.0	0.64
Chronic disease (%)	55.3	56.0	59.8	50.6	0.004
Nutrient data					
Energy intake (kcal/d)	1927.7 ± 658.4	1810.7 ± 604.3	1871.6 ± 651.8	2085.4 ± 682.0	<0.001
Carbohydrate intake (g/day)	240.5 ± 93.8	240.9 ± 91.1	230.2 ± 87.7	249.4 ± 100.6	0.001
Total fat intake (g/d)	72.7 ± 33.6	61.9 ± 30.2	71.0 ± 33.3	84.0 ± 33.4	<0.001
Protein intake (g/d)	73.9 ± 30.3	76.3 ± 29.9	73.9 ± 32.2	71.6 ± 28.7	0.02
Alcohol intake (g/d)	8.0 ± 21.7	4.7 ± 11.7	7.5 ± 18.6	11.4 ± 29.5	<0.001
Dietary fiber (g/d)	16.0 ± 9.4	21.5 ± 10.9	15.6 ± 7.9	11.5 ± 6.0	<0.001
Carbohydrate intake (%E)	50.4 ± 11.1	53.6 ± 10.7	49.8 ± 11.0	47.9 ± 10.9	<0.001
Total fat intake (%E)	33.5 ± 9.2	30.3 ± 8.7	33.7 ± 9.0	36.4 ± 9.0	<0.001
Protein intake(%E)	15.6 ± 4.7	17.1 ± 4.6	16.0 ± 4.7	14.0 ± 4.1	<0.001
Anthropometric and body composition					
BMI (kg/m^2^)	27.7 ± 4.6	27.1 ± 4.4	28.1 ± 4.6	27.8 ± 4.6	0.001
ASMI (kg/m^2^)	7.26 ± 1.31	7.10 ± 1.29	7.27 ± 1.33	7.40 ± 1.31	<0.001
FFMI (kg/m^2^)	17.80 ± 2.57	17.42 ± 2.51	17.83 ± 2.60	18.11 ± 2.57	<0.001
FMI (kg/m^2^)	10.09 ± 3.41	9.93 ± 3.30	10.46 ± 3.37	9.89 ± 3.52	0.005
Total body fat (%)	35.5 ± 7.7	35.7 ± 7.6	36.4 ± 7.4	34.6 ± 8.0	<0.001
Strength					
Peak force (Newtons)	377.0 ± 121.9	367.9 ± 119.5	369.8 ± 121.9	391.8 ± 122.9	<0.001
Peak torque (Newton meter)	117.9 ± 43.1	114.5 ± 42.1	115.5 ± 42.7	123.1 ± 44.0	<0.001
Time to peak force (seconds)	1.09 ± 0.58	1.14 ± 0.76	1.09 ± 0.50	1.05 ± 0.42	0.01
Angle of peak force (degree)	122.3 ± 7.2	122.0 ± 7.6	122.6 ± 6.8	122.3 ± 7.3	0.37
Peak force velocity (degree/second)	60.7 ± 0.6	60.6 ± 0.7	60.7 ± 0.6	60.7 ± 0.6	0.05

^a^Most anti-inflammatory values of the E-DII

^b^Most pro-inflammatory values of the E-DII

Individuals with higher E-DII scores were predominantly male and younger than average. The percentage of individuals with more than high school education decreased from 61.1% in the lowest E-DII tertile to 47.5% in the highest tertile. The percentage of no smokers decreased from 53.5% in the lowest E-DII tertile to 39.2% in the highest tertile; in contrast, the percentage of current smokers increased from 6.6% in the lowest tertile to 25.0% in the highest tertile. For dietary intake, the individuals who had higher E-DII scores consumed more calories, carbohydrate, fat, and alcohol, less protein and fiber. The individuals with higher E-DII scores presented with higher FFMI, ASMI and muscle strength (PF, PT); and lower FMI and body fat percentage.

## The association of ASMI, peak force with demographic characteristics and lifestyle and nutrient

The results of univariate analysis were shown in Supplementary Table 2. The results of univariate analysis showed that former smoker, heavy work, BMI, energy intake and protein intake were positively correlated with ASMI and PF. We also found that age and female sex were inversely associated with ASMI and PF.

## The relationship between E-DII and muscle mass and strength

We used a multiple linear regression model to evaluate the associations between E-DII score and ASMI and PF. Logistic regression models were used to examine the association between E-DII score and low muscle mass and low muscle strength, alone and combined. Table 2 shows the results of the crude (non-adjusted) and adjusted models. As seen in Table 2 for muscle mass, in the crude model, the *β* of ASMI was 0.08 (95% confidence interval (CI): 0.05 to 0.10, *P* < 0.001). We detected an association in the fully adjusted model (*β* =  −0.03, 95% CI  −0.04 to −0.01, *P* < 0.001). Furthermore, we also performed a sensitivity analysis, analyzing the E-DII score as a categorical variable (tertile), and found the same trend across tertiles (*P* for the trend was <0.001). As seen in Table 2 for muscle strength, in the crude model, the *β* of PF was 6.33 (95% confidence interval (CI) 3.67 to 8.99, *P* < 0.001). We detected a negative association between DII and PF in the fully adjusted model (*β* =  −2.15, 95% CI  −4.19 to −0.12, *P* = 0.04) and found the same trend across tertiles (*P* for the trend was 0.01). As seen in Table 2, each one-unit increase in DII was both positively associated with a 9% increase in low muscle mass and low muscle strength in the fully adjusted logistic model (OR 1.09, 95% CI 1.01–1.17 for low muscle mass; OR 1.09, 95% CI 1.01–1.18 for low muscle strength). Each one-unit increase in E-DII was positively associated with a 12% increase in combined low muscle mass and low muscle strength in the fully adjusted logistic model (OR 1.12, 95% CI 1.01–1.25, Table 2).

**Table 2:** Associations between E-DII and muscle mass and muscle strength in different models^a^

Variable	Crude model		Model 1^b^		Model 2^c^	
	*β*/OR (95%CI)	*P*-value	*β*/OR (95%CI)	*P*-value	*β*/OR (95%CI)	*P*-value
ASMI (kg/m^2^)						
E-DII	0.08 (0.05, 0.10)	<0.001	−0.02 (−0.04, −0.01)	<0.001	−0.03 (−0.04, −0.01)	<0.001
E-DII						
T1	Reference		Reference		Reference	
T2	0.13 (−0.01, 0.28)	0.07	−0.10 (−0.17, −0.04)	0.001	−0.11 (−0.17, −0.04)	0.001
T3	0.30 (0.15, 0.44)	<0.001	−0.12 (−0.18, −0.05)	<0.001	−0.13 (−0.20, −0.06)	<0.001
*P* for trend	<0.001		<0.001		<0.001	
Low muscle mass^d^						
E-DII	1.01 (0.96, 1.07)	0.65	1.07 (1.00, 1.15)	0.04	1.09 (1.01, 1.17)	0.02
E-DII						
T1	Reference		Reference		Reference	
T2	1.36 (1.02, 1.80)	0.03	1.42 (1.04, 1.95)	0.03	1.450 (1.05, 2.00)	0.02
T3	1.08 (0.81, 1.44)	0.60	1.39 (1.00, 1.93)	0.05	1.452 (1.03, 2.06)	0.03
*P* for trend	0.61		0.05		0.03	
Peak force (Newtons)						
E-DII	6.33 (3.67, 8.99)	<0.001	−1.43 (−3.37, 0.51)	0.15	−2.15 (−4.19, −0.12)	0.04
E-DII						
T1	Reference		Reference		Reference	
T2	2.59 (−10.37, 15.56)	0.69	−10.22 (−19.31, −1.13)	0.03	−11.47 (−20.65, −2.29)	0.01
T3	24.03 (11.07, 37.00)	<0.001	−9.34 (−18.68, −0.004)	0.05	−12.12 (−21.81, −2.43)	0.01
*P* for trend	<0.001		0.05		0.01	
Low muscle strength^e^						
E-DII	0.99 (0.93, 1.05)	0.64	1.06 (0.99, 1.14)	0.10	1.09 (1.01, 1.18)	0.02
E-DII						
T1	Reference		Reference		Reference	
T2	0.98 (0.74, 1.30)	0.88	1.10 (0.79, 1.54)	0.56	1.16 (0.82, 1.63)	0.40
T3	0.98 (0.74, 1.30)	0.88	1.40 (0.99, 1.99)	0.06	1.56 (1.08, 2.25)	0.02
*P* for trend	0.88		0.06		0.02	
Low muscle mass and Low muscle strength^f^						
E-DII	1.04 (0.95, 1.14)	0.38	1.12 (1.01, 1.24)	0.03	1.12 (1.01, 1.25)	0.03
E-DII						
T1	Reference		Reference		Reference	
T2	1.16 (0.75, 1.79)	0.51	1.29 (0.80, 2.07)	0.29	1.29 (0.80, 2.08)	0.30
T3	1.24 (0.81, 1.90)	0.33	1.66 (1.02, 2.71)	0.04	1.68 (1.01, 2.79)	0.045
*P* for trend	0.33		0.04		0.045	

^a^*E-DII* energy-adjusted Dietary Inflammatory Index; *ASMI* appendicular skeletal muscle index; *CI* confidence interval

T1, T2, T3—E-DII is divided into three groups

^b^Model 1 adjusted for age (years), sex, race, education, marital status, nativity, smoking, physical activity level and BMI

^c^Model 2 adjusted for Model I + Chronic disease, Energy and Protein

^d^Low muscle mass was defined using the Foundation for the National Institute of Health definitions: ASM divided by BMI (men <0.789; women <0.512)

The multiple regression explored the association between E-DII (independent variable) and low muscle mass (dependent variable), and both Model I and Model II did not adjust for BMI, and the remaining adjustments remained the same

^e^Low muscle strength were defined as the peak torque value that fell more than 2 SDs below the sex-specific mean peak torque of this healthy 50–59-year-old reference group (men <104.4 Nm; women <62.6 Nm)

^f^A combination of low muscle mass and low muscle strength was used as a representation of sarcopenia. Model 1 adjusted for age (years), sex, race, education, marital status, nativity, smoking and physical activity level; Model 2 adjusted for Model 1 + Chronic disease, Energy and Protein

## Sensitivity analysis and subgroup analyses

In view of vitamin D as an important confounding factor, after further adjustment of serum vitamin D, whether the effect value in the multiple linear regression model is still stable. Vitamin D data were available in the 2001–2002 cycle, but this was not measured during the 1999–2000 cycle; thus, the sample size was reduced to 893. The multiple linear regression model showed that after adjusting for age, sex, race, education, marital status, nativity, smoking, physical activity level, BMI, chronic disease status, energy, protein and serum 25(OH)D, E-DII and ASMI (the dependent variable) were still negatively correlated (*β* = −0.03, *P* = 0.01, *P* for trend = 0.01).In the fully adjusted multiple linear regression model, the *β* of PF was −2.46 (compared to the *β* −2.15 in Table 2), and it could be observed that the direction and magnitude of the effect value remained stable overall; however, this was not statistically significant. Compared with the lowest tertile of E-DII, the *β* of PF was −13.99 (95% CI −27.71, −0.27) in the top tertile (*P* for trend <0.05).

To further confirm that the findings observed in Table 2 are robust to potential confounders, we performed stratified analyses by subgroups defined by major covariates known to affect ASMI and PF. As shown in Supplementary Table 4, the dependent variables are ASMI and PF, respectively. The tests for interaction were not statistically significant for sex, race, chronic disease status, energy intake, protein energy ratio, serum micronutrients (vitamin A, vitamin E, *α*-tocopherol, total carotenoid, *β*-carotene and vitamin D) and fasting insulin level (*P* values for all interactions were larger than 0.05).

There was evidence of an interaction between E-DII score and low muscle mass. The effect sizes of E-DII on PF showed significant differences in low muscle mass (yes/no). E-DII was negatively associated with PF in low muscle mass (*β* =  −5.58, 95%CI  −9.92,  − 1.24).

## Discussion

The risk of low muscle mass /sarcopenia has become a major concern for quality of life in the context of aging populations and in individuals with various chronic disease [42, 43]. Also, muscle mass is inversely associated with the risk of death [44]. To the best of our knowledge, this is the first study to investigate the association between DII/ E-DII and muscle mass and strength in older US adults. We found that the E-DII score was negatively associated with ASMI and peak force in older adults. A more pro-inflammatory diet, as indicated by higher E-DII score, increased the likelihood for low muscle mass, low muscle strength and the combination of low muscle mass and low muscle strength.

Inflammation has been proven to contribute to muscle depletion by both triggering protein breakdown and impairing myogenesis [45]. Pro-inflammatory cytokines, including TNF-*α*, IL-1, IL-6 and IFN-*γ*, impinge on muscle protein metabolism. The Health, Aging, and Body Composition (Health ABC) study, conducted on 3075 Black and White men and women aged 70–79 years, confirmed that higher concentrations of IL-6 and TNF-*α* are associated with lower muscle mass and lower muscle strength in well-functioning older persons [19]. The results of a study of 4984 adults aged ≥60 years from NHANES 1999–2004 also revealed significant inverse associations between appendicular lean mass (ALM) adjusted for body mass index (ALM_BMI_) and CRP and fibrinogen [46]. A systematic review and meta-analysis showed that higher levels of CRP, IL-6 and TNF-α were associated with lower handgrip and knee extension strength [47]. The inflammatory cytokines produced in the context of chronic inflammation may influence their respective related pathways, thus leading to loss of muscle mass, strength, and function [20].

Inflammation is closely related to skeletal muscle insulin resistance. Inflammation primarily impairs insulin signaling through the activation of the IKK*β*/NF-*κ*B and JNK pathways, thereby promoting skeletal muscle insulin resistance [48, 49]. Theoretically, loss of muscle mass leads to a decrease in insulin-mediated glucose metabolism in the body, which can lead to insulin resistance. In contrast, insulin resistance may contribute to skeletal muscle loss because insulin promotes protein synthesis as well as glucose uptake. A cross-sectional study in non-diabetic older adults examined the association of muscle mass and muscle strength with insulin resistance, and verified that lower-limb muscle mass is related to insulin resistance independent of obesity. However, muscle strength (knee extension torque) was not significantly related to insulin resistance [50]. Our results show that lower skeletal muscle mass is independently associated with insulin resistance in older adults, which is consistent with Lee et al.’s [51] findings. A combined retrospective–prospective cohort study of 147 community-dwelling older men and women participants showed insulin resistance was associated with low relative ASM at 4.6-year follow-up [52]. Insulin resistance assessed by HOMA has been associated with a pronounced loss of ASM in one longitudinal cohort study [53].

Diet is an important modulator of chronic systemic inflammation [24]. The DII was developed to assess the role of diet in relation to health outcomes ranging from inflammatory cytokine concentrations in the blood to chronic diseases [38, 54]. The E-DII is an important refinement that often produces higher levels of explanatory ability than does the DII [54]. Single nutrients and the DII have been validated against inflammatory biomarkers [55–57]. Two studies carried out construct validation of the DII with C-reactive protein (CRP) utilizing data came from NHANES. Both studies showed a positive association between the DII and CRP in a nationally representative sample [27, 58]. Our results showed that the E-DII also was associated with CRP ≥3 mg/l (OR DII continuous = 1.06; 95% CI 1.01, 1.12) in participants aged ≥50 years. The effects of specific components of DII on muscle mass and strength have been reported in some studies. Dietary fat profile, which has been associated with cardiovascular disease protection, may be beneficial for conservation of skeletal muscle mass [59, 60]. In a cross-sectional study included older adults aged from 50 to 85 years from the National Health and Nutrition Examination Survey (NHANES) 1999–2002, the intake of total ω-3 fatty acids was positively associated with muscle strength in older men, but not in older women [37]. Considering that single nutrients or foods may not be able to describe overall dietary effects or may focus on the wrong individual effects, the advantage of the DII/E-DII score is that it considers interactions and intercorrelations among foods and nutrients, instead of examining individual nutrients or foods in relation to disease.

Our research results show that higher E-DII score (indicating a more pro-inflammatory diet) was associated with lower ASMI, indicating lower muscle mass. In 466 Chinese children aged 6–9 years, the survey revealed that the DII score was inversely associated with skeletal muscle mass in boys but not in girls [61]. In a cross-sectional study that recruited 599 individuals aged 18–25 years in Spain, linear regression analysis revealed that fat-free mass (FFM) was significantly associated with the DII (β =  −0.059; 95% CI  −0.842, −0.107; *P* = 0.01) [62]. The two studies in older Australians have reported similar results. In a cross-sectional study of 809 participants aged 60–95 years from the Geelong Osteoporosis Study, after adjusting for covariates, higher DII score was associated with lower ALM/height^2^ [28]. In a cross-sectional analysis of 794 community-dwelling older men, a higher E-DII score also was associated with lower appendicular lean mass adjusted for BMI (ALM _BMI_) (β =  −0.006 kg/m^2^) [18]. Together, these findings highlight the ability of the inflammatory potential of diet to influence the maintenance of skeletal muscle mass across the life course, and that pro-inflammatory diets can cause poor musculoskeletal health through a variety of mechanisms [18].

Another finding of our research was that higher E-DII score is associated with lower muscle strength. In the updated 2018 definition, EWGSOP2 uses low muscle strength as the primary indicator of probable sarcopenia [31]. The examination of muscle strength mainly includes measuring grip strength and chair stand test. When measurement of grip is not possible, isometric torque methods can be used to measure lower limb strength [63]. In a study of 321 community-dwelling individuals aged 70–85 years in Korea, DII score was positively associated with the risk of low walking speed, and low grip strength [64]. In a cross-sectional study of 300 elderly people aged ≥55 years in Iran, no significant association was seen between DII and abnormal handgrip strength and abnormal gait speed [30]. DII scores in this Iranian study population ranged from −3.98 to 4.29, while the DII scores in our study ranged from −5.79 to 4.39. The range of DII in a prospective study in the Tasmanian Older Adult Cohort Study (TASOAC) with 1098 men and women aged 50–79 years was −3.80 to +3.23 and increased DII score was not associated with knee extensor strength, whole lower-limb muscle strength, or handgrip strength [29]. To our knowledge, this is the first study to investigate the association between the inflammatory potential of the diet and the knee extensor strength in elderly Americans. The inconsistency of research results may be due to different methods used to assess muscle strength, limited DII score ranges, and other unmeasured factors. Therefore, more high-quality research is required to determine the effects of diet-associated inflammation on muscle strength in older adults.

The presence of low muscle strength and low muscle quantity confirmed the diagnosis of sarcopenia [31]. This study showed that higher E-DII score was associated with a higher likelihood for a combination of low muscle mass and low muscle function (i.e., a representation of sarcopenia). A cross-sectional study of 300 Iranians aged 55 years or older revealed that a diet with more pro-inflammatory potential was associated with a greater odds of sarcopenia [30]. A cross-sectional study of 809 older Australians showed that the higher DII score was associated with increased likelihood for the combination of low muscle mass (low ALM/height^2^)and low muscle function(Timed-Up-and-Go >10 s over 3 m) [28]. Our findings are in agreement with those two cross-sectional studies. In a study of 1098 Australian older adults in the TASOAC Study, the associations between E-DII scores and changes in sarcopenia-related outcomes from baseline to 10 years were not significant in either men or women [29]. However, due to several factors, it is difficult to directly compare this result with ours. E-DII score ranges in longitudinal TASOAC study were limited; i.e., ranged from −3.80 to +3.23 units. In our study, the DII/E-DII range is wider. The different criteria were used to define sarcopenia. In our study, a combination of low muscle mass and low muscle strength was used as a representation of sarcopenia. Low muscle mass was defined as ASM/BMI <0.789 for men and <0.512 for women, and low muscle strength were defined as the peak torque value (men <104.4 Nm; women <62.6 Nm). Because ethnicity, body size, lifestyles and culture differ between European and Asian populations, there is no consensus about the definition of sarcopenia criteria. Our research is a cross-sectional study, and further prospective studies investigating whether pro-inflammatory dietary interventions could increase the risk of sarcopenia are needed.

This study has several strengths. A validated dietary assessment method was used to generate the E-DII scores. Objective measures were used to assess muscle mass and muscle strength. Our adjustment strategy included a large number of factors associated with skeletal muscle. A sensitivity analysis was conducted to classify muscle mass and muscle strength (continuous variable) as low muscle mass and low muscle strength (yes/no), and a logistic regression model was used to evaluate the association between E-DII (predictor) and low muscle mass and low muscle strength and the combination (outcome).

Despite these strengths, there are also some limitations to acknowledge. First, because of the cross-sectional nature of the study, the association between the E-DII score and muscle mass and strength could not speak to the temporal criterion of judging causality [65]. A prospective experimental research design is more suitable to addressing this issue. Second, the observational design of this study could not exclude the possibility of residual confounding caused by unknown risk factors. Third, the BMI of individuals included in this study is ≥18.5, and the standard of BMI <18.5 cannot be used; only the low fat-free mass index (FFMI) is used, and the unintentional weight loss is not taken into account because this information is not included in the database. Fourth, dietary intake was estimated based on one 24HR conducted during the survey, which may not reflect habitual dietary intake. Using only 1 day of dietary information may not explain the day-to-day variability in diet, thus resulting in inaccurate estimates [66]. The 24-h dietary recall method has inevitable misreporting bias. Fifth, individuals with higher E-DII scores were predominantly male and younger, and the plasma level of testosterone and insulin are essential to explain the association between diet, muscle mass and strength. Regrettably, at the moment at the time of writing and publishing this article it was not possible to access the testosterone data from NHANES because the NHANES website was under maintenance. Although our study results showed that an elevated insulin concentration is associated with increases of ASM, a prospective cohort study design is needed, which would allow for measuring the risk of developing loss of ASM in relation serum insulin after a follow-up period. Also, the role of reverse causality is unascertainable; i.e., how changes in healthy behaviors (such as diet and physical activity) may be affected by feelings of well-being or ill health. These, in turn, may affect the inflammatory state and its association with musculoskeletal health.

In conclusion, the E-DII score was negatively associated with ASMI and Peak force, revealing that a higher E-DII score was related to lower muscle mass, lower muscle strength and increased likelihood for the combination of low muscle mass and low muscle strength in older US adults. These findings suggest that a more pro-inflammatory diet (higher DII score) negatively affects muscle mass and muscle strength, and reducing the consumption of a more pro-inflammatory diet reduces the risk of sarcopenia. Further well-designed studies are needed to clarify these associations, and confirm whether anti-inflammatory dietary interventions can reduce the risk of sarcopenia.

## Supplementary Information

Below is the link to the electronic supplementary material.

Supplementary file1 (DOCX 60 KB)

## Abbreviations

ASMI: Appendicular skeletal muscle index
ASM: Appendicular skeletal muscle mass
ALM: Appendicular lean mass
ALM_BMI_: Appendicular lean mass adjusted for BMI
CAD: Coronary artery disease
CHF: Congestive heart failure
CVD: Cardiovascular disease
DII®: Dietary inflammatory index
DEXA: Dual-energy X-ray absorptiometry
E-DII: Energy-adjusted dietary inflammatory index
EWGSOP: European Working Group on Sarcopenia in Older People
FFM: Fat-free mass
FFMI: Fat-free mass index
hs-CRP: High-sensitivity C-reactive protein
MEC: Mobile examination center
NHANES: National Health and Nutrition Examination Survey
PF: Peak force
PT: Peak torque
TASOAC: Tasmanian Older Adult Cohort Study
TNF-α: Tumor necrosis factor-α
24HR: 24-Hour dietary recall

## Declarations

### Clinical trial registry

Not applicable, as this was based on an analysis of existing observational data from the NHANES.
