# Supplementary material for: Association between dietary inflammatory index score and muscle mass and strength in older adults: a study from National Health and Nutrition Examination Survey (NHANES) 1999–2002
Source: Eur J Nutr. 2022 Jul 9;61(8):4077–89. doi: 10.1007/s00394-022-02941-9 (PMC9596556; doi:10.1007/s00394-022-02941-9)
Supplement: Supplementary file 1 — Supplementary file1 (DOCX 60 KB) [file 394_2022_2941_MOESM1_ESM.docx]

**Association between dietary inflammatory index score and muscle mass and strength in older adults: A study from National Health and Nutrition Examination Survey (NHANES) 1999-2002**

**Author:** Jingjing Ming , Lingzhi Chen

Excluded participants

no data for body composition measure

(*n* = 9,908)

Participants interviewed in the

NHANES 1999–2000, 2001–2002 datasets

(*n* = 21,004)

Participants with body composition measure data

(*n* = 11,096)

Total excluded participants (*n* = 9,233)

• BMI <18.5kg/m^2^ (*n* = 1,257)

• Missing E-DII score (*n* = 265)

• Total energy intake outside of the predeﬁned limits (*n*=1,126)

•Muscle strength test aged <50 years (*n* = 5,690)

extreme values of peak force velocity (*n* = 882)

<4 trials (*n* = 13)

Overall study population (*n* = 1,863)

**Fig. 1** Participant flowchart from the population of older adult participants in the NHANES 1999–2002

**Supplementary Table 1** Correlation coefficients for E-DII and nutrient intake (total energy, macronutrients, ω -3 fatty acids), National Health and Nutrition Examination Survey (NHANES) 1999-2002

|  | E-DII score | Energy intake | Protein intake | Carbohydrate intake | Total fat intake | ω-3 fatty acids intake |
| --- | --- | --- | --- | --- | --- | --- |
| E-DII score | 1.00 | - | - | - | - | - |
| Energy intake | 0.16 | 1.00 | - | - | - | - |
| Protein intake | -0.05 | 0.71 | 1.00 | - | - | - |
| Carbohydrate intake | 0.02 | 0.82 | 0.43 | 1.00 | - | - |
| Total fat intake | 0.29 | 0.80 | 0.59 | 0.43 | 1.00 | - |
| ω-3 fatty acids intake | -0.07 | 0.48 | 0.39 | 0.26 | 0.59 | 1.00 |

**Supplementary Table 2** Crude association of ASMI , PF with demographic characteristics , lifestyle ,and nutrient variables, National Health and Nutrition Examination Survey (NHANES) 1999-2002

|  |  |  |  |  |  |  |  |  |  |  |  |  |  |
| --- | --- | --- | --- | --- | --- | --- | --- | --- | --- | --- | --- | --- | --- |
| Characteristic | | | | | Statistics | | | | β (95%CI) | | *P* -value | | |
| **ASMI**, kg/m^2^ | | | | |  | | | |  | |  | | |
| Age (year) | | | | 64.71 ± 9.78 | | | | -0.03 (-0.03, -0.02) | | | | <0.001 |  |
| Sex | | | |  | | | |  | | | |  |  |
| male | | | | 990 (53.14%) | | | | ref | | | |  |  |
| female | | | | 873 (46.86%) | | | | -1.63 (-1.72, -1.54) | | | | <0.001 |  |
| Race/ethnicity | | | |  | | | |  | | | |  |  |
| Non-Hispanic White | | | | 1084 (58.19%) | | | | ref | | | |  |  |
| Others | | | | 779 (41.81%) | | | | 0.30 (0.18, 0.42) | | | | <0.001 |  |
| Education | | | |  | | | |  | | | |  |  |
| Less than high school | | | | 646 (34.73%) | | | | ref | | | |  |  |
| High School Diploma | | | | 415 (22.31%) | | | | -0.18 (-0.34, -0.03) | | | | 0.02 |  |
| More than high school | | | | 799 (42.96%) | | | | 0.01 (-0.12, 0.14) | | | | 0.89 |  |
| Marital status | | | |  | | | |  | | | |  |  |
| unmarried | | | | 518 (29.05%) | | | | ref | | | |  |  |
| married | | | | 1265 (70.95%) | | | | 0.52 (0.39, 0.65) | | | | <0.001 |  |
| Nativity | | | |  | | | |  | | | |  |  |
| foreign-born | | | | 368 (19.77%) | | | | ref | | | |  |  |
| US-born | | | | 1493 (80.23%) | | | | -0.07 (-0.21, 0.08) | | | | 0.37 |  |
| Smoking | | | |  | | | |  | | | |  |  |
| No | | | | 854 (45.94%) | | | | ref | | | |  |  |
| Former smoker | | | | 726 (39.05%) | | | | 0.50 (0.37, 0.62) | | | | <0.001 |  |
| Current smoker | | | | 279 (15.01%) | | | | 0.13 (-0.04, 0.30) | | | | 0.14 |  |
| Physical activity level | | | |  | | | |  | | | |  |  |
| Sits | | | | 424 (22.84%) | | | | ref | | | |  |  |
| Walks | | | | 1068 (57.54%) | | | | -0.16 (-0.31, -0.02) | | | | 0.03 |  |
| Light loads | | | | 280 (15.09%) | | | | -0.09 (-0.28, 0.11) | | | | 0.38 |  |
| Heavy work | | | | 84 (4.53%) | | | | 0.41 (0.10, 0.71) | | | | 0.01 |  |
| BMI (kg/ m^2^) | | | | 27.69 ± 4.41 | | | | 0.17 (0.16, 0.18) | | | | <0.001 |  |
| Chronic disease | | | |  | | | |  | | | |  |  |
| No | | | | 730 (39.57%) | | | | ref | | | |  |  |
| Yes | | | | 1115 (60.43%) | | | | 0.12 (-0.001, 0.24) | | | | 0.05 |  |
| Energy intake (kcal/d) | | | 1854.1±644.7 | | | 0.0004(0.0003, 0.0005) | | | | | <0.001 | |  |
| Energy intake (kcal/d) | | | |  | | | |  | | | |  |  |
| <1800 | | | | 949 (50.94%) | | | | ref | | | |  |  |
| ≥1800 | | | | 914 (49.06%) | | | | 0.51 (0.40, 0.63) | | | | <0.001 |  |
| Protein intake (g/d) | | | | 71.86 ± 30.06 | | | | 0.01 (0.007, 0.011) | | | | <0.001 |  |
| Protein,% of energy | | | |  | | | |  | | | |  |  |
| <15% | | | | 924 (49.60%) | | | | ref | | | |  |  |
| ≥15% | | | | 939 (50.40%) | | | | 0.10 (-0.02, 0.21) | | | | 0.11 |  |
| **Peak force** , Newtons | | | | |  | | | |  | |  | | |
| Age (year) | | | | 64.71 ± 9.78 | | | | -4.98 (-5.47, -4.49) | | | | <0.001 |  |
| Sex | | | |  | | | |  | | | |  |  |
| male | | | | 990 (53.14%) | | | | ref | | | |  |  |
| female | | 873 (46.86%) | | | | | -129.7 (-138.6, -120.9) | | | | | <0.001 |  |
| Race/ethnicity | | | |  | | | |  | | | |  |  |
| Non-Hispanic White | | | | 1084 (58.19%) | | | | ref | | | |  |  |
| Others | | | | 779 (41.81%) | | | | -2.07 (-12.84, 8.70) | | 0.71 | | |  |
| Education | | | |  | | | |  | | | |  |  |
| Less than high school | | | | 646 (34.73%) | | | | ref | | | |  |  |
| High School Diploma | | | | 415 (22.31%) | | | | 12.21 (-2.09, 26.51) | | 0.09 | | |  |
| More than high school | | | | 799 (42.96%) | | | | 35.62 (23.60, 47.65) | | | | <0.001 |  |
| Marital status | | | |  | | | |  | | | |  |  |
| unmarried | | | | 518 (29.05%) | | | | ref | | | |  |  |
| married | | | | 1265 (70.95%) | | | | 56.84 (45.21, 68.47) | | | | <0.001 |  |
| Nativity | | | |  | | | |  | | | |  |  |
| foreign-born | | | | 368 (19.77%) | | | | ref | | | |  |  |
| US-born | | | | 1493 (80.23%) | | | | 10.67 (-2.67, 24.02) | | | | 0.12 |  |
| Smoking | | | |  | | | |  | | | |  |  |
| No | | | | 854 (45.94%) | | | | ref | | | |  |  |
| Former smoker | | | | 726 (39.05%) | | | | 42.63 (31.22, 54.04) | | | | <0.001 |  |
| Current smoker | | | | 279 (15.01%) | | | | 22.15 (6.56, 37.73) | | | | 0.005 |  |
| Physical activity level | | | |  | | | |  | | | |  |  |
| Sits | | | | 424 (22.84%) | | | | ref | | | |  |  |
| Walks | | | | 1068 (57.54%) | | | | -4.00 (-17.08, 9.08) | | | | 0.55 |  |
| Light loads | | | | 280 (15.09%) | | | | 13.54 (-4.01, 31.09) | | | | 0.13 |  |
| Heavy work | | | | 84 (4.53%) | | | | 62.33 (35.12, 89.55) | | | | <0.001 |  |
| BMI (kg/ m^2^) | | | | 27.69 ± 4.41 | | | | 5.25 (4.07, 6.43) | | | | <0.001 |  |
| Chronic disease | | | |  | | | |  | | | |  |  |
| No | | | | 730 (39.57%) | | | | ref | | | |  |  |
| Yes | | | | 1115 (60.43%) | | | -27.44 (-38.30, -16.58) | | | | | <0.001 |  |
| Energy intake (kcal/d) | | | 1854.1±644.7 | | | 0.05 (0.04, 0.06) | | | | | <0.001 | |  |
| Energy intake (kcal/d) | | | |  | | | |  | | | |  |  |
| <1800 | | | | 949 (50.94%) | | | | ref | | | |  |  |
| ≥1800 | | | | 914 (49.06%) | | | | 57.00 (46.69, 67.31) | | | | <0.001 |  |
| Protein intake (g/d) | | | | 71.86 ± 30.06 | | | | 0.90 (0.73, 1.07) | | | | <0.001 |  |
| Protein,% of energy | | | |  | | | |  | | | |  |  |
| <15% | | | | 924 (49.60%) | | | | ref | | | |  |  |
| ≥15% | | | | | 939 (50.40%) | | | | 0.93 (-9.70, 11.56) | | 0.86 | | |

**Supplementary Table 3** Multiple linear regression coefficients of muscle mass and strength indicators with homeostasis model assessment of insulin resistance, National Health and Nutrition Examination Survey (NHANES) 1999-2002

| Variable | ASM | | |  | ASMI | | | |  | Peak Force | | |  | Peak Torque | | | |  |
| --- | --- | --- | --- | --- | --- | --- | --- | --- | --- | --- | --- | --- | --- | --- | --- | --- | --- | --- |
|  | β(95%CI) | *P*-value | |  | β (95%CI) | | *P*-value | |  | β (95%CI) | *P*-value | |  | | β (95%CI) | *P*-value | | |
| Crude Model | 0.11 (0.05, 0.17) | <0.001 | |  | 0.03 (0.02, 0.05) | | <0.001 | |  | 0.28 (-1.12, 1.69) | | 0.69 |  | 0.18 (-0.32, 0.68) | | | 0.48 |  |
| Model 1 | -0.03 (-0.06, -0.01) | | 0.003 |  | | -0.01 (-0.01, 0.003) | | 0.21 |  | -1.16 (-2.15, -0.17) | | 0.02 |  | -0.47 (-0.79, -0.15) | | | 0.004 |  |
| Model 2 | -0.03 (-0.05, -0.005) | | 0.02 |  | -0.01 (-0.02, -0.002) | | | 0.02 |  | -1.12 (-2.11, -0.12) | | 0.03 |  | -0.39 (-0.70, -0.08) | | | 0.01 |  |

896 participants were tested for fasting serum insulin and fasting glucose and were included in the data analysis

Insulin resistance was measured by HOMA-IR, which was calculated as the product of fasting glucose (mmol/L) and fasting insulin (μU/ML) divided by 22.5.

Model 1: adjusted for sex, age, weight. Model 2: adjusted for sex, age, weight, height, smoking, alcohol intake, and C-reactive protein.

**Supplementary Table 4** Effect size of E-DII on ASMI (kg/m^2^) and Peak force (Newtons) in exploratory subgroups,^a^ National Health and Nutrition Examination Survey (NHANES) 1999-2002

|  |  | | | | | | |
| --- | --- | --- | --- | --- | --- | --- | --- |
| Characteristic | | No of participants | β (95%CI) | | *P* -value | | *P* for  interaction |
| **ASMI** , kg/m^2^  Sex | |  |  | |  | |  |
| male | | 990 | -0.02 (-0.05, -0.004) | | 0.02 | | 0.78 |
| female | | 873 | -0.03 (-0.05, -0.01) | | 0.006 | |  |
| Race/ethnicity | |  |  | |  | |  |
| Non-Hispanic White | | 1084 | -0.03 (-0.05, -0.01) | | 0.002 | | 0.18 |
| Others | | 779 | -0.01 (-0.04, 0.01) | | 0.34 | |  |
| Chronic disease | |  |  | |  | |  |
| NO | | 730 | | -0.03 (-0.05, -0.005) | 0.02 | | 0.49 |
| YES | | 1115 | -0.03 (-0.05, -0.01) | | 0.01 | |  |
| Energy intake, kcal/d | |  |  | |  | |  |
| <1800 | | 949 | | -0.02 (-0.04, 0.003) | 0.10 | | 0.37 |
| ≥1800 | | 914 | -0.03 (-0.05, -0.01) | | 0.004 | |  |
| Protein,% of energy | |  |  | |  | |  |
| <15% | | 924 | -0.02 (-0.04, 0.003) | | 0.09 | | 0.71 |
| ≥15% | | 939 | -0.03 (-0.05, -0.01) | | 0.001 | |  |
| Malnutrition | |  |  | |  | |  |
| No | | 1495 | -0.02 (-0.04, -0.01) | | 0.006 | | 0.81 |
| Yes | | 368 | -0.03 (-0.05, -0.005) | | 0.02 | |  |
| Physical activity , MET min/week ^b^ | |  |  | |  | |  |
| <500 | | 306 | -0.02 (-0.06, 0.02) | | 0.26 | | 0.78 |
| 500–1000 | | 220 | -0.002 (-0.05, 0.04) | | 0.93 | |  |
| ≥1000 | | 496 | -0.02 (-0.04, 0.01) | | 0.16 | |  |
| Vitamin A (ug/dL) | |  |  | |  | |  |
| Low(<64.9) | | 447 | -0.02 (-0.05, 0.01) | | 0.17 | | 0.35 |
| High(≥64.9) | | 452 | -0.03 (-0.06, 0.001) | | 0.06 | |  |
| Vitamin E (ug/dL) | |  |  | |  | |  |
| Low(<1461.1) | | 449 | -0.01 (-0.04, 0.02) | | 0.41 | | 0.46 |
| High(≥1461.1) | | 450 | -0.04 (-0.07, -0.01) | | 0.01 | |  |
| α-Tocopherol (ug/dL) | |  |  | |  | |  |
| Low(<1218.5) | | 446 | -0.01 (-0.05, 0.02) | | 0.36 | | 0.60 |
| High(≥1218.5) | | 447 | -0.04 (-0.07, -0.01) | | 0.01 | |  |
| Total carotenoids (ug/dL) | |  |  | |  | |  |
| Low(<73.7) | | 444 | -0.01 (-0.04, 0.03) | | 0.63 | | 0.37 |
| High(≥73.7) | | 447 | -0.04 (-0.07, -0.01) | | 0.01 | |  |
| β-Carotene (ug/dL) | |  |  | |  | |  |
| Low(<18.7) | | 448 | -0.00 (-0.03, 0.03) | | 0.98 | | 0.10 |
| High(≥18.7) | | 449 | -0.04 (-0.06, -0.01) | | 0.01 | |  |
| Vitamin D( nmol/L) | |  |  | |  | |  |
| Low(<58.7) | | 399 | -0.05 (-0.08, -0.02) | | | 0.002 | 0.05 |
| High(≥58.7) | | 494 | -0.01 (-0.03, 0.02) | | 0.68 | |  |
| Fasting insulin (uU/mL) ^c^ | |  |  | |  | |  |
| Low(<10.2) | | 448 | -0.02 (-0.05, 0.01) | | 0.14 | | 0.93 |
| High(≥10.2) | | 449 | -0.02 (-0.05, 0.01) | | 0.23 | |  |
| Low muscle strength | |  |  | |  | |  |
| NO | | 1513 | | -0.02 (-0.03, -0.001) | | 0.03 | 0.06 |
| YES | | 350 | -0.05 (-0.08, -0.02) | | 0.002 | |  |
| **Peak force** , Newtons | |  |  | |  | |  |
| Sex | |  |  | |  | |  |
| male | | 990 | -2.60 (-5.77, 0.58) | | 0.11 | | 0.80 |
| female | | 873 | -1.61 (-3.98, 0.77) | | 0.18 | |  |
| Race/ethnicity | |  |  | |  | |  |
| Non-Hispanic White | | 1084 | -3.08 (-5.69, -0.46) | | 0.02 | | 0.07 |
| Others | | 779 | -0.14 (-3.43, 3.15) | | 0.93 | |  |
| Chronic disease | |  |  | |  | |  |
| NO | | 730 | -3.38 (-6.49, -0.27) | | 0.03 | | 0.21 |
| YES | | 1115 | -1.32 (-4.01, 1.37) | | 0.33 | |  |
| Energy intake, kcal/d | |  |  | |  | |  |
| <1800 | | 949 | -1.61 (-4.42, 1.19) | | 0.26 | | 0.92 |
| ≥1800 | | 914 | -1.88 (-4.77, 1.01) | | 0.20 | |  |
| Protein,% of energy | |  |  | |  | |  |
| <15% | | 924 | -3.57 (-6.34, -0.79) | | 0.01 | | 0.12 |
| ≥15% | | 939 | -0.70 (-3.62, 2.22) | | 0.64 | |  |
| Malnutrition | |  | |  |  | |  |
| No | | 1495 | | -1.06 (-3.37, 1.25) | 0.37 | | 0.25 |
| Yes | | 368 | | -4.01 (-7.71, -0.30) | 0.03 | |  |
| Physical activity,  MET min/week ^b^ | |  | |  |  | |  |
| <500 | | 306 | | -0.59 (-5.52, 4.34) | 0.81 | | 0.92 |
| 500–1000 | | 220 | | -0.85(-7.52, 5.83) | 0.80 | |  |
| ≥1000 | | 496 | | -1.91 (-5.74, 1.93) | 0.33 | |  |
| Vitamin A (ug/dL) | |  |  | |  | |  |
| Low(<64.9) | | 447 | -1.48 (-5.37, 2.40) | | 0.45 | | 0.47 |
| High(≥64.9) | | 452 | -2.75 (-7.34, 1.85) | | 0.24 | |  |
| Vitamin E (ug/dL) | |  |  | |  | |  |
| Low(<1461.1) | | 449 | -0.04 (-4.50, 4.43) | | 0.99 | | 0.24 |
| High(≥1461.1) | | 450 | -3.59 (-7.78, 0.60) | | 0.09 | |  |
| α-Tocopherol (ug/dL) | |  |  | |  | |  |
| Low(<1218.5) | | 446 | -1.35 (-5.91, 3.21) | | 0.56 | | 0.63 |
| High(≥1218.5) | | 447 | -3.09 (-7.16, 0.97) | | 0.14 | |  |
| Total carotenoids (ug/dL) | |  |  | |  | |  |
| Low(<73.7) | | 444 | -4.06 (-8.76, 0.64) | | 0.09 | | 0.22 |
| High(≥73.7) | | 447 | -1.20 (-5.20, 2.80) | | 0.56 | |  |
| β-Carotene (ug/dL) | |  |  | |  | |  |
| Low(<18.7) | | 448 | -2.33 (-7.24, 2.58) | | 0.35 | | 0.87 |
| High(≥18.7) | | 449 | -2.86 (-6.71, 0.98) | | 0.14 | |  |
| Vitamin D( nmol/L) | |  |  | |  | |  |
| Low(<58.7) | | 399 | -2.44 (-7.08, 2.21) | | 0.30 | | 0.67 |
| High(≥58.7) | | 494 | -1.88 (-5.80, 2.05) | | 0.35 | |  |
| Fasting insulin (uU/mL) ^c^ | |  | |  |  | |  |
| Low(<10.2) | | 447 | | -5.25(-9.58, -0.93) | 0.02 | | 0.48 |
| High(≥10.2) | | 449 | | -2.04 (-6.44, 2.37) | 0.36 | |  |
| Low muscle mass | |  | |  |  | |  |
| NO | | 1502 | | -1.09 (-3.33, 1.15) | 0.34 | | 0.03 |
| YES | | 361 | | -5.58 (-9.92, -1.24) | 0.01 | |  |

^a^ Above model adjusted for age (years), sex, race, education, marital status, nativity, smoking, physical activity level , BMI, Chronic disease, Energy and Protein.

In each case, the model is not adjusted for the stratification variable.

Number of samples: Vitamin A (*n*=899), Vitamin E (*n*=899), α-Tocopherol (*n*=893), Total carotenoids (*n*=891), β-Carotene (*n*=897) and Vitamin D (*n*=893).

^b^ 1022 participants had physical activity (MET min/week) data and were included in the data analysis. Adjusted for age (years), sex, race, education, marital status, nativity, smoking, BMI, Chronic disease, Energy, Protein and Malnutrition.

^c^ Fasting serum insulin data were available for 897 participants and included in the data analysis.
